# Jazz improvisers' shared understanding: a case study

**DOI:** 10.3389/fpsyg.2014.00808

**Published:** 2014-08-08

**Authors:** Michael F. Schober, Neta Spiro

**Affiliations:** ^1^Department of Psychology, New School for Social ResearchNew York, NY, USA; ^2^Centre for Music and Science, Faculty of Music, University of CambridgeCambridge, UK

**Keywords:** shared understanding, intersubjectivity, collaboration, communication, interaction, improvisation, music, jazz

## Abstract

To what extent and in what arenas do collaborating musicians need to understand what they are doing in the same way? Two experienced jazz musicians who had never previously played together played three improvisations on a jazz standard (“It Could Happen to You”) on either side of a visual barrier. They were then immediately interviewed separately about the performances, their musical intentions, and their judgments of their partner's musical intentions, both from memory and prompted with the audiorecordings of the performances. Statements from both (audiorecorded) interviews as well as statements from an expert listener were extracted and anonymized. Two months later, the performers listened to the recordings and rated the extent to which they endorsed each statement. Performers endorsed statements they themselves had generated more often than statements by their performing partner and the expert listener; their overall level of agreement with each other was greater than chance but moderate to low, with disagreements about the quality of one of the performances and about who was responsible for it. The quality of the performances combined with the disparities in agreement suggest that, at least in this case study, fully shared understanding of what happened is not essential for successful improvisation. The fact that the performers endorsed an expert listener's statements more than their partner's argues against a simple notion that performers' interpretations are always privileged relative to an outsider's.

## Introduction

When musicians play together, they predict, perceive, and react to what their partners do in complex ways. Doing this requires some level of shared understanding—but of what exactly? In what arenas and to what extent do collaborating musicians creating a joint performance need to understand what they are doing in the same way? And to what extent is their understanding privileged—more shared—relative to the understanding of a non-performing listener?

At a minimum (or even by definition) it must be the case that the musicians agree that music is happening. For most kinds of music it is also essential that they have overlapping (though not necessarily fully identical) understandings of a rhythmic structure, or else no joint performance would be possible. But must *everything* be agreed upon? How much discrepancy in understanding can there be in a successful joint performance? That is, to what extent do collaborating musicians agree on every aspect of musical structure, or their concept of the piece, or each other's musical intentions at each moment, or their judgment of the quality of the performance?

Joint musical performance is, of course, a particular case of joint action that has its own particular and genre-specific qualities (Keller, 2008), and one could ask variants of these questions in other domains of interaction. In studying conversation, for example, one could ask to what extent conversational partners' mental representations of linguistic and conceptual structure are identical (e.g., Pickering and Garrod, 2004; Schober, 2005), or to what extent they understand the activity and agendas of the conversation to be the same (e.g., Forgas, 1983; Russell and Schober, 1999), or to what extent they accurately judge each other's intentions at different moments [whether they accurately recognize the other's referring expressions (Clark and Wilkes-Gibbs, 1986; Garrod and Anderson, 1987), or the other's ironic intent or implied criticism or emotional state (e.g., Ickes, 1993)], or to what extent they agree in their judgments of whether the conversation was successful or enjoyable or irritating. In a grand sense all these questions are related to aspects of partners' *common ground* (Clark, 1996)—their knowledge, beliefs and assumptions about what they mutually know. But exactly how common ground builds and what is relevant in conversation clearly differs in musical interaction, which usually does not have referential content or the kinds of communicative intention that are found in conversations (Cross, in press), even if musical interaction can have conversation-like back-and-forth structures (e.g., Monson, 1996; Healey et al., 2005).

The aim in this case study was to explore the extent to which a pair of experienced jazz musicians understand what they have done together in the same way: whether they spontaneously generate the same descriptions of their performances and intentions, and even when they do not, whether they agree with their partner's characterization of what happened and what was intended. The methodological approach was to collect immediate retrospective accounts by performers after their performances, and then to examine the extent to which either party later endorsed the statements that they themselves and their partner had made, as well as statements made by an expert listener (see Gottman and Levenson, 1985; Brown and Pavlicevic, 1996, and Ickes et al., 1986, for examples of this approach in other domains of interaction).

Our approach differs from what has been done thus far in empirical studies of music performance in our explicit focus on (1) how performers linguistically articulate their understanding of what has happened musically and on (2) the extent to which performers agree with each other's articulations. Behavioral and neuroscientific observation of musicians playing together (e.g., Clayton, 2005; Keller et al., 2007; Goebl and Palmer, 2009; Kirschner and Tomasello, 2009; Luck and Sloboda, 2009; Pecenka and Keller, 2009; Keller and Appel, 2010; Novembre et al., 2012; among many others) can allow inferences about the shared mental representations (explicit or implicit) underlying joint performance—for example, how players mentally represent their partners, what kinds of anticipatory and monitoring mechanisms must be in effect for coordinated music-making to work, or how perception of a partner's motion affects synchronization. But they do not often get at musicians' own characterizations of these mental representations—of their mental life while playing.

Although we interview musicians, our approach also differs in focus from prior interview studies that analyze musicians' more general reflections on, for example, how they feel about their musical roles (King, 2006), their relationships with other performers (Myers and White, 2012), their sense of empathy in peak performances (Waddington, 2013), the nature of improvisation (Sawyer, 1992), or their identity as a musician (MacDonald and Wilson, 2005). We focus instead on musicians' detailed accounts immediately after specific performances because, like Davidson and Good (2002), we are interested in how performers understand their moment-by-moment collaboration: how they believe they put forward musical ideas, how they hear those ideas as taken up or rejected, when and if they impute intention to their co-performers, etc. And we are interested in the extent to which these understandings are shared.

Rather than examining how the musicians communicate with each other (e.g., Davidson and King, 2004; Ginsborg and King, 2012), we are interested in exploring players' musical thinking *independent* of their co-performers' thinking. Of course musicians rehearsing and playing together do talk about the music, their interpretations, and their roles, and so one would expect (if the partnership persists) that at least some characterizations of what has happened musically are likely to become more shared over time and with more and more conversation (Williamon and Davidson, 2002). In the current study we focus on one pair's initial musical encounter, and we interview them about their understanding of what happened without allowing them to discuss their performance at all; in fact, we never let them talk to each other or even see each other (The situation is thus more akin to playing with strangers in a recording session booth than to a musical partnership that develops face to face with the usual casual and task-oriented conversation that allows musicians to get to know each other as performers and as social beings).

As we see it, the extent to which co-performers agree or disagree with each other's characterizations is likely to vary substantially in different pairings, different performances, and for different kinds of statements. We would expect that competent musicians should agree, given their ear and experience, on basic musicological facts about what happened—who started the piece, what key they were in, how many verses or choruses were played (if it's that sort of piece), and when their rhythms and tempi matched or mismatched. But it is less clear whether players will agree in their evaluations of the quality or flavor of a particular performance, in their judgment of what their partner meant by a particular musical gesture, in when the collaboration was more and less smooth, or in how responsibility is assigned (for example, who is leading at any particular moment). As Wöllner (2013) demonstrated in a string quartet, quartet members who rated (on a continuous slider) their own and each others' expressivity while watching and listening to video of an earlier joint performance did not necessarily agree with each other, in either direction or valence (see also Seddon, 2005).

In the current study, we selected the genre of jazz improvisation, and we gave the performers the task of playing a jazz standard together three times in a row, in a different way of their choosing each time. Given the genre, we expected that the performers might afterwards especially talk about the collaborative aspects of improvisation: making offers, picking up on the other's gestures or hints, sensing that the partner was ready to change gears, etc. We also were aware of the possibility that, given the free flow and interdependence of improvisation, performers might find it difficult to articulate precise moments when a single thing had happened or where they had made conscious or specific musical judgments. But our intention in the study was to start with what the performers themselves thought worth articulating, and even though we used focused prompts to encourage them to talk, to try as much as possible to elicit *their* perspectives on what they had done.

For the performers' likelihood of later endorsing each other's characterizations, various patterns of results are possible. If both players independently were to generate all the same statements, or if they did not but they were to fully endorse all each other's statements, this would demonstrate strong intersubjective agreement. If they were to endorse each other's statements more than they endorsed an outside listener's, this would suggest that the co-performers hold a privileged understanding, perhaps similar to the way that speakers and addressees in a dialog tend to understand each other's referring expressions better than eavesdroppers do (Schober and Clark, 1989). If players do not later endorse even the statements they themselves made, this would suggest that interpretations are fleeting and unstable, whether because they are not memorable or because understandings change. If players only endorse some of their partner's characterizations (while continuing to endorse their own), the extent of that endorsement and the kinds of statements that they disagree about should be informative about what kinds of understandings they share.

## Materials and methods

### Participants

The performing participants were a professional jazz saxophonist and a professional jazz pianist who had never previously met. Both were male, one in his 20s and the other in his 30s, and thus of the same general cohort; both regularly perform in New York City, and both are graduates of (different) leading jazz performance programs. They each were compensated $100 for participating two times, first on the day of performance and then for a second phase of data collection.

The outside listener who provided additional characterizations of the performances was a faculty member at The New School for Jazz and Contemporary Music (Dan Greenblatt) who has published on jazz performance and theory (e.g., Greenblatt, 2004, 2013). He performs professionally and has recorded as a jazz saxophonist, and he has extensive experience on panels evaluating student performances.

Informed consent for participating and for releasing summary results was obtained from all participants, and consent for releasing audio recordings of the performances (available in Supplementary Materials) was obtained from the performers, following review of the procedure by the New School's Institutional Review Board.

### Materials

#### Day of performance

A list of potential pieces for performance was generated consisting of 9 jazz standards that the performers might feel comfortable improvising with an unfamiliar partner. Tunes were selected in order to be (a) challenging enough to keep the interest of two good players through three interpretations; (b) flexible enough to provide a range of improvisatory options, for example having common alternate chord changes and not traditionally being played in a single standard key; and (c) common enough so that both performers would have a high likelihood of knowing them and being comfortable playing them. The list (*Here's That Rainy Day*, *Embraceable You*, *It Could Happen to You*, *You Stepped Out of a Dream*, *How Deep is the Ocean*, *Green Dolphin Street*, *Day by Day*, *If I Should Lose You*, *Old Folks*) was printed on two sheets of paper, with the instruction “Please circle the tunes you know well enough to feel comfortable playing in a duo context with a very good jazz saxophonist” (or “pianist”).

Audio recordings of the performances were burned to CDs and immediately provided for presentation to the players on a laptop during the interviews.

A set of prompt questions and interviewing suggestions was visible to both interviewer and performer throughout the discussion. A first set of general discussion prompts was intended to stimulate statements from memory about the differences between the performances. A second set of prompts was intended to focus commentary while listening (potentially multiple times, with stops and starts controlled by the performer being interviewed) to the recordings that had just been made. The full set of prompts is presented in Table 1.

**Table 1:** **Interview prompts**.

**General discussion prompts**	**Prompts during listening**
How would you describe the differences in the three performances you two just gave?	What do you think worked and what didn't?
	How did you know what to do next?
What was the difference in character between these three performances? (Ask if not already covered by response to first question)	What did your partner do that struck you as particularly interesting or notable?
Were there any differences in the quality of performance? (Ask if not already covered by response to first question)	Did anything strike you as particularly notable about your own performance?
How easy or hard was it to play with your partner? Why (Please be as specific as you can)? Did this change over time?	Were there any moments that you had trouble playing together? Do you remember when they were?
What did your partner do that struck you as particularly interesting or notable? (Please be as specific as you can, and about in which version)	Can you point at particular musical choices you made that your partner picked up on? That your partner didn't pick up on?
Did anything strike you as particularly notable about your own performance? (Please be as specific as you can, and about in which version)	Did your partner make particular musical choices that you recognized while you were playing? That you rejected?
Were there any moments that you had trouble playing together? Do you remember when they were?	
Can you describe, as specifically as possible, how you reached agreement—for each version—on how to start, how to structure the piece (e.g., improvising or not, soloing or not, how many choruses), and how to end the piece?	
Did you feel that someone was in charge? Did this change during your performances? Was it different in the different versions? How did you know?	
Can you point at particular musical choices you made that your partner picked up on? That your partner didn't pick up on?	
Did your partner make particular musical choices that you recognized while you were playing? That you picked up on or rejected?	

#### Retrospective rating

A questionnaire was constructed (by both authors) consisting of statements made about the performances by the players themselves in the interviews immediately after the performances (70 by the pianist and 34 by the saxophonist) as well as 64 additional statements by the expert outside listener. The outside listener's statements were made in writing soon after the performances in response to the same recordings; he had been shown the prompts used in the interviews with the performers. Of the set of statements, 28 were judged as alternate versions of the same claim made by two parties, with 12 statements made by all three parties; for example, the pianist's “He started the second song,” the sax player's “I started playing first” (referring to the second performance), and the expert's “Here the sax takes the lead from the start” were judged to be sufficiently similar to be considered alternate versions of the same claim. The 28 two-party and 12 three-party statements were summarized in new joint wordings (e.g., “The sax started this performance”), creating 14 and 4 statements, respectively. One additional statement (“The sax was more responsible than the pianist for the quality of this take”) was created as a transformation of the original statement “The pianist was more responsible than the sax for the quality of this take.” This procedure resulted in 151 unique statements that could be rated.

The statements were anonymized and made consistent so that the original author of the statements could not be ascertained and to point to the moment in the recording that the performer was discussing; for example, “I was playing a bass part” was transformed into “At about 4:08 the sax plays a bass part”; the statements about the sax player's starting the second performance were transformed into “The sax started this performance.” Of the 151 statements, 114 were specific statements that could only apply to one performance (e.g., “Just before about 0:28, the sax was waiting for the piano to play the tonic as a cue to start the melody”); 33 were generalizable statements—made about one performance but potentially applicable to any of the three performances (e.g., “The overall performance was standard or ‘vanilla’”; “The sax started this performance”); and 4 were global—general statements about the players and performances (e.g., “My partner's signals were very clear”). Although the outside listener produced a few statements that used particularly technical musicological language (e.g., “The pianist substitutes at bar 15-16 (around 0:52) (“down you tumble”) as | F#-7 B7 | F-7 Bb7 | rather than | F-7 | Bb7 | ”; “At about 0:17 the piano changes the quality to Phrygian, signaling a more functional dominant”), all three included technical language and jazz-specific terminology (see **Figures 5A–E** for the full set of anonymized statements with attribution of source).

**Figure 1:**
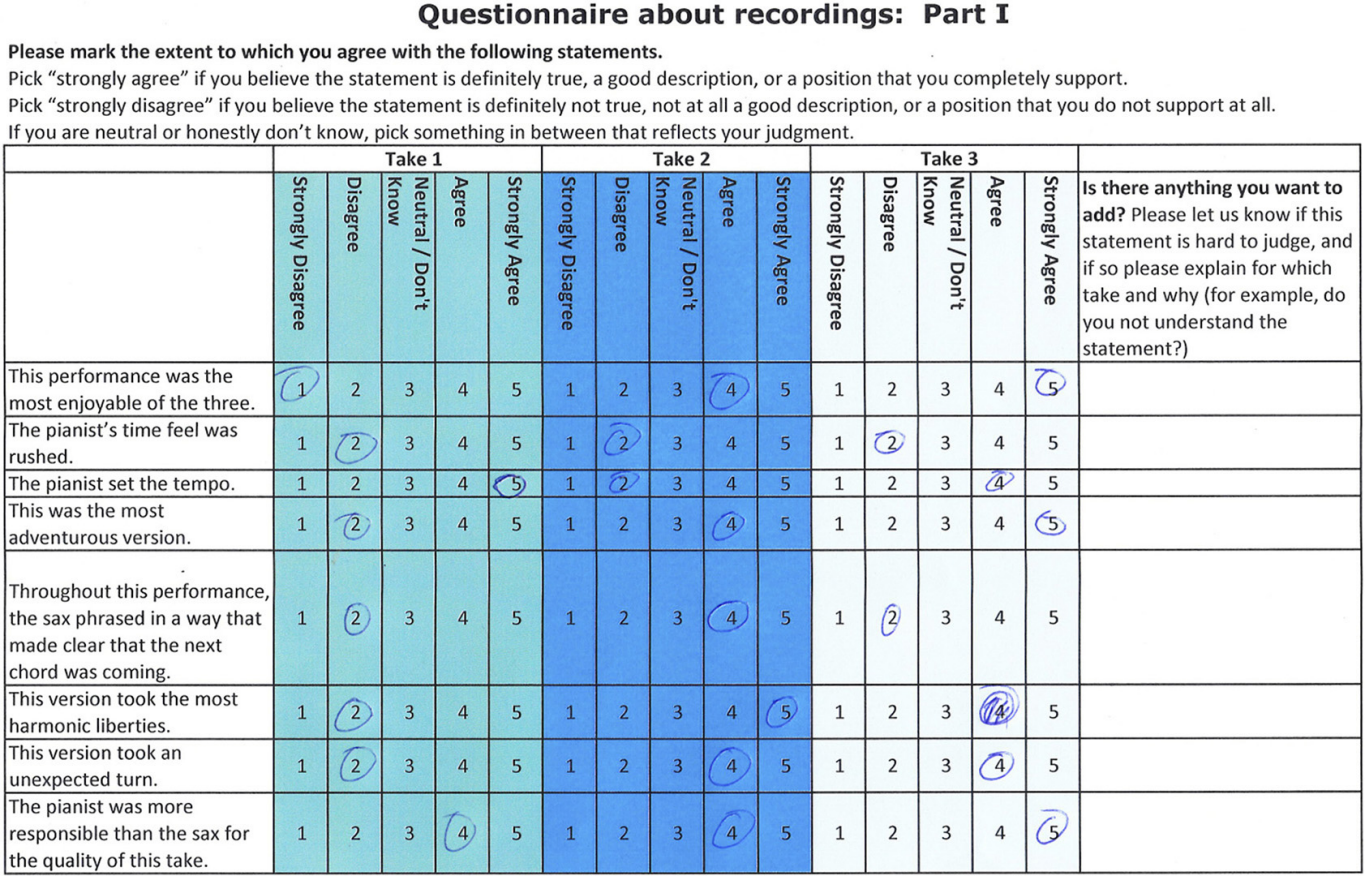
**Start of questionnaire part (b)—generalizable statements that could apply to all three performances**.

The questionnaire presented each statement for rating on a five point scale from Strongly Disagree (1) to Strongly Agree (5). The questionnaire consisted of five parts: (a) global statements about players and performances; (b) generalizable statements that could apply to more than one performance; (c) specific statements that concerned just one performance; and (d) questions about the experience of completing the questionnaire (e.g., “Did you find yourself remembering how you felt at the time or thinking about how you feel about things now when listening or a bit of both?”). For statements in (b), the questionnaire asked participants to rate the extent to which they endorsed each statement for each of the three performances (see Figure 1) so that the questionnaire ended up requiring 217 separate ratings of the statements about the performances.

The statements in parts (a) and (b) were presented in a random order and the questions in part (c) were presented in the order corresponding to the moments in the recording to which they applied.

### Procedure

#### Day of performance

Throughout the day, the performers never heard each other's names, met each other, saw each other, or heard the other speak; they entered and exited and they were briefed and debriefed entirely separately.

**Figure 2:**
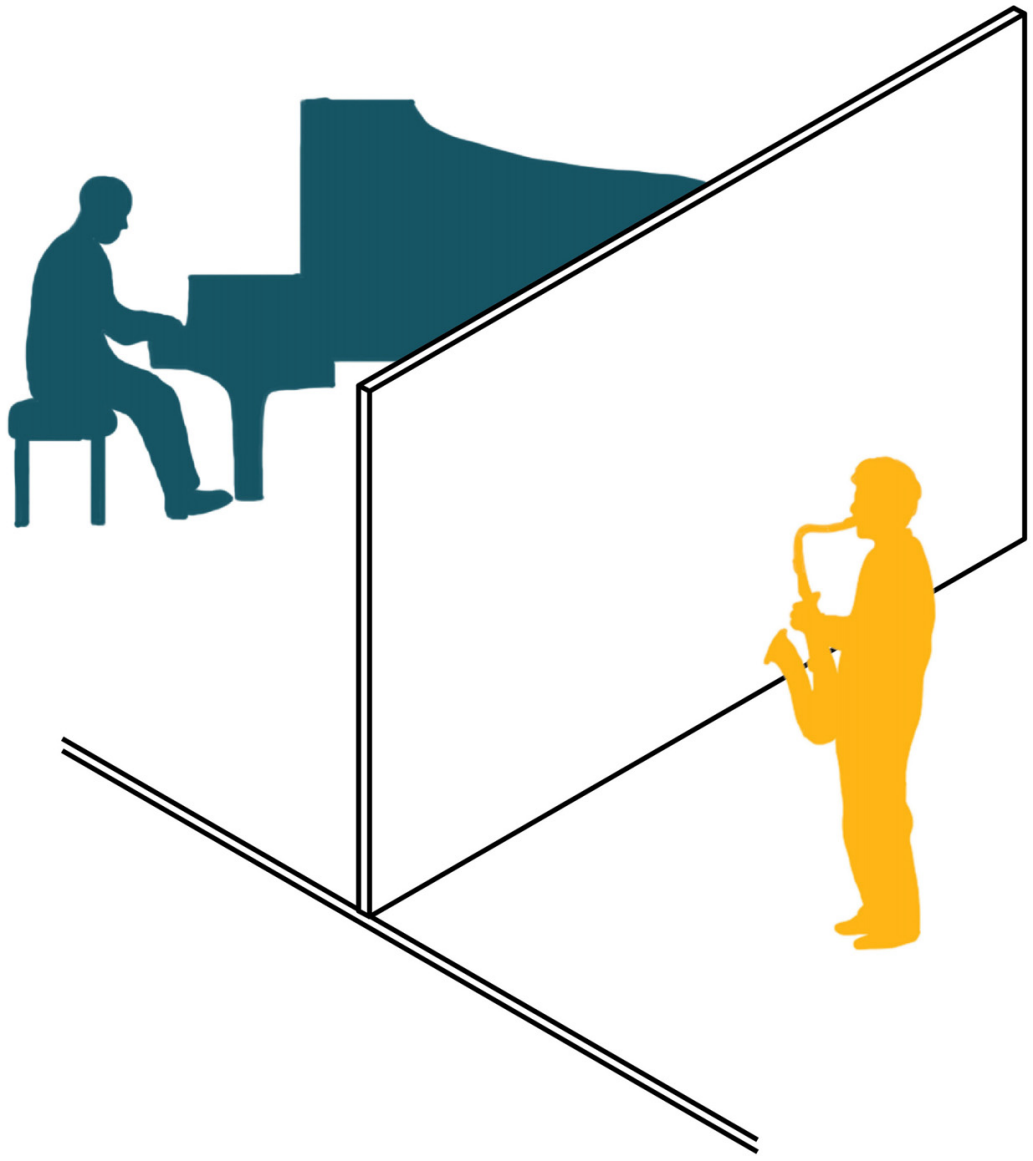
**Positioning of pianist and sax player, on either side of a screen onstage in the performance space**.

After the performers entered the experiment room, which was a performance space at the New School for Jazz and Contemporary Music in New York City, they were seated on either side of a visual barrier with the piano on one side (see Figure 2) and then asked to select (on paper) which standards they would be comfortable performing (see Materials). The experimenter selected “It Could Happen to You” from those on the list that overlapped, and then presented instructions to both performers simultaneously. The performers were asked to improvise three versions of “It Could Happen to You” that should be different from each other and that should each last about 5 min and be separated by silence; the performers were never to speak to each other at all, neither before, during or after the performances. The performances were audiorecorded using the performance space's high quality microphones and recording equipment, and immediately burned to CDs (Recordings of the three performances are available in Supplementary Materials).

Immediately after the performances, each player was interviewed separately by different interviewers (the first author and a psychology graduate student) about the three performances. Both interviewers had experience carrying out research interviews, musical training, and experience playing in the instrument group about which they were interviewing (piano or brass) in other genres, and non-expert levels of experience with jazz performance. Interviews were carried out using the prompts (see Materials), first soliciting the players' general observations about the performances and then their observations prompted by listening to recordings of the performances. The interviewers' task was to elicit detailed and specific commentary that addressed the target questions, in whatever way seemed appropriate for each performer; the interviewers were instructed to be flexible and to probe further for any statements that could be clearer or more specific about which moment in the music they were referring to. For the General Discussion prompts, interviewers were instructed to probe if players were not forthcoming (“Can you say more?” “Were there any other differences?” “Did anything else strike you?” etc.), and to request clarification if a player said anything the interviewer didn't understand. For the prompted listening, interviewers first played each recording the whole way through before using the prompts to elicit commentary; after that the players were encouraged to start and stop the recording themselves as often as they liked to make specific comments in the spirit of the prompts. The interviewers were encouraged to probe further if they did not understand which precise moment or section in a recording the player was referring to. The interviews, which were audiorecorded for subsequent use, took about one hour each.

#### Retrospective rating

Two months later, the performers were given access to MP3s of the three recordings and paper-and-pencil hardcopies of the questionnaire, for them to fill out at their convenience in a quiet place alone, without interruption. (The interval of two months reflected time for questionnaire development, and it was intended to minimize the risks of both immediate retrospective biases and long-term forgetting. Other time intervals would, no doubt, create different measurement biases). They were instructed to listen to each recording at least once with headphones before responding, and that they could listen to each recording, with starts, stops, and rewinds, as often as they liked in order to provide accurate ratings. They were told that we were interested in their responses as they listened to the recordings *now*, and that this was not intended to be a memory test of how they felt at the time of recording. Both performers returned the questionnaires within a week; they each reported having taken about an hour to complete the questionnaires. The performers at this point were still uninformed of the other's identity.

The outside listener also carried out the same ratings. The questionnaires and materials were identical except for a few minor necessary modifications of statement wording (e.g., turning statements that contained “my partner” into two statements, one about “the sax player” and one about “the pianist”). In addition, the listener was asked to rate the quality of the performances based on his experience as a jury member for a jazz conservatory.

#### Subsequent elaboration

After the retrospective ratings were returned, we followed up with each player individually for further elaboration on exactly what each dissented about for the statements on which the players clearly disagreed (by 3 or 4 rating points). In order to keep burden to a minimum, participants were offered the possibility to respond in writing, in a telephone interview, or in person as they wished given their schedules and preferences. The saxophonist elaborated about his dissents in writing via email, and the pianist about his dissents in a telephone interview, which was audiorecorded and transcribed. We also asked each player to tell us about his history performing “It Could Happen to You”; both reported performing it quite frequently in their professional lives.

## Results

### Overlap in performers' statements

To what extent did the players independently generate the same statements? There was only a small amount of overlap—less than 10%—in the players' characterizations of what had happened: 8 of the 104 statements made by both players were made “spontaneously” by both parties. Each of the 8 statements that both players generated turned out also to have been generated by the outside listener. This means that, in fact, there were no statements that were exclusively generated by only the performers.

We can't, of course, take the interview material as a reliable indicator of everything that the players had been thinking; the interviews were carried out by two different interviewers (so that they could happen simultaneously and immediately after the performance), and we cannot know whether different interviewers might have elicited different observations or characterizations. Nor do we know how differently each player might have characterized the performances on a different occasion even with the same interviewers. Nonetheless, it is notable that the overlap was low, given that the prompt questions were uniform. Clearly what the performers thought worthy of articulating was substantially different, and their (few) overlapping observations were not unique relative to an outsider.

### Ratings of statements

#### Specificity of endorsements

Because the 33 generalizable statements allow a within-subjects comparison of each player's ratings for the three performances, we could test whether the patterns of endorsement were specific to the performances the statements were originally about. As Table 2 shows, the evidence is that the endorsements were indeed quite specific; the average level of endorsement was significantly higher when ratings were about the original performance than when the same statements were rated for the other performances, *F*_(4, 60)_ = 8.60, *p* < 0.001 (from an ANOVA with performance and player as repeated-measures factors, and whether the rating was about the original performance as a between-group factor). This increases our confidence that the ratings were thoughtful and the statements specific to the performances and moments to be rated.

**Table 2:** **Average endorsement (both players) for generalizable statements**.

	**Ratings about**		
	**Performance 1**	**Performance 2**	**Performance 3**
**STATEMENT ORIGINALLY ABOUT**			
Performance 1	**3.8**	2.0	2.8
Performance 2	2.1	**4.2**	2.3
Performance 3	3.1	3.2	**3.8**

Ratings about the original performance are in bold.

#### Endorsing own vs. others' statements

**Figure 3:**
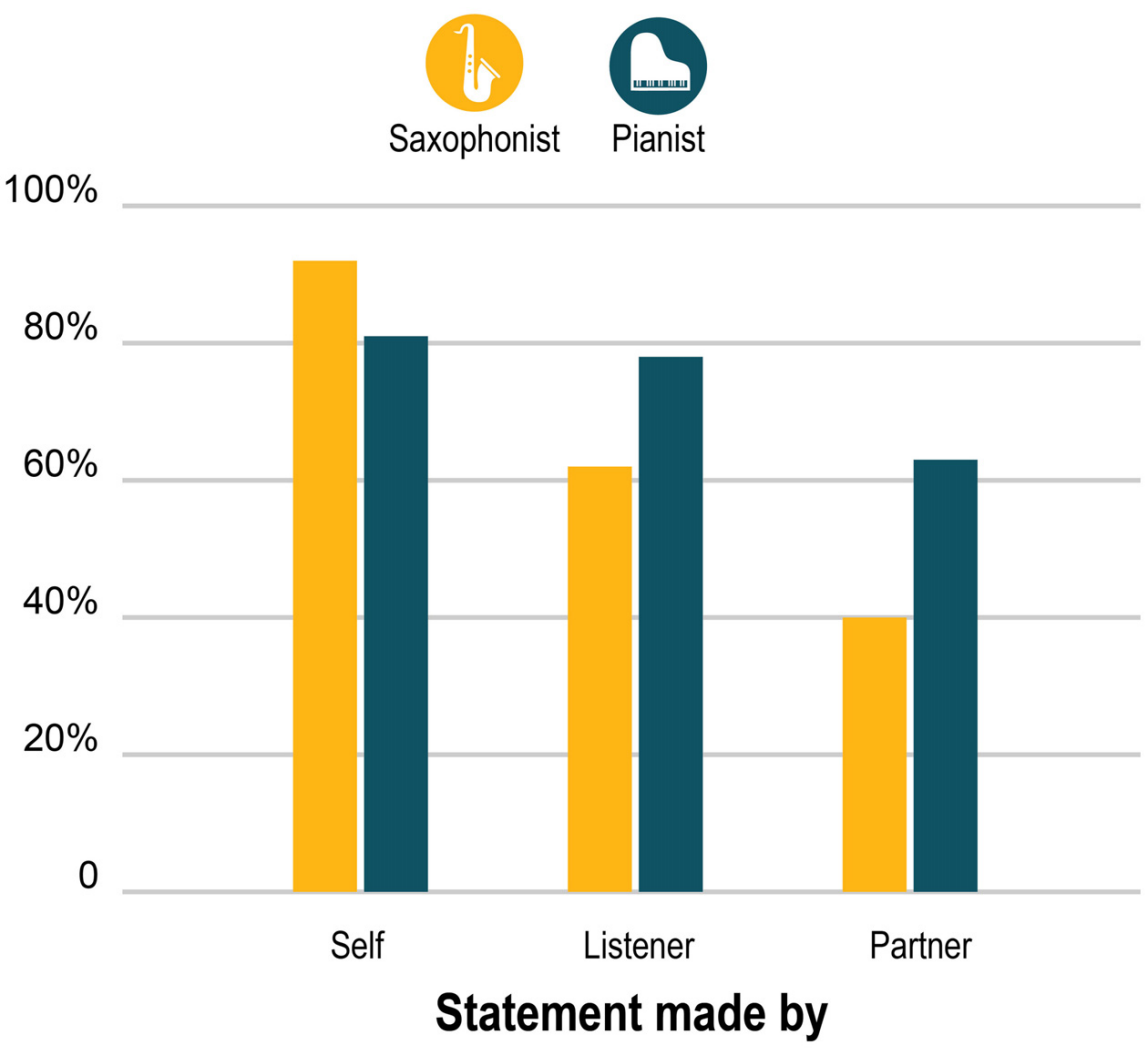
**Percent of the 151 statements originally made by themselves, the outside listener, and their partner that the pianist and saxophonist endorsed (by selecting 4 or 5 on the 5-point scale)**.

Both performers endorsed a strong majority—though not all—of the (anonymized) statements that they themselves had generated two months earlier. As Figure 3 shows, both performers endorsed their own statements more often than statements by the outside listener, and they endorsed the outside listener's statements more than the statements by their performing partner. The reductions in endorsement of the outside listener's and the partner's statements were greater for the sax player than for the pianist, who endorsed nearly as great a percentage of the statements by the listener (a sax expert) as his own. But the pattern steps down in a strikingly similar way for both players.

Of course, we cannot rule out the possibility that part of performers' endorsing their own statements was related to their wanting to be consistent in endorsing statements they recognized as their own, or in recognizing that they had *not* made a statement, even though two months had elapsed since the original interviews and even though the statements had been anonymized. Nonetheless, the relative ranking of endorsements of the listener's and partner's judgments cannot be accounted for by this feature of the method.

#### Agreement between the players

To quantify the extent to which both players agreed in endorsing or dissenting with statements made by each party, we calculated Cohen's kappa to measure inter-rater agreement for all 217 ratings. We also included their agreement with the outside listener's ratings as a point of comparison (note that using kappa to test levels of agreement is quite different from the usual usage of kappa to validate a coding scheme and ratings by testing inter-coder reliability). Table 3 shows kappas calculated using three ratings categories: endorsement (4 or 5 on the 5-point scale), neutral (3), and dissent (1 or 2 on the 5-point scale).

**Table 3:** **Inter-rater agreement (Cohen's kappa), calculated with three categories: both endorse, both neutral, both dissent (Categories with too few statements for kappa to be calculated are not reported)**.

	**Number of statements**	**Sax-pianist agreement**	**Sax-listener agreement**	**Pianist-listener agreement**
Overall	217	**0.280**	0.378	0.320
**STATEMENT MADE BY**				
Saxophonist	62	**0.355**	0.583	0.282
Pianist	90	**0.178**	0.311	0.203
Outside listener	50	**0.335**	0.063	0.203
**PERFORMANCE THAT WAS RATED**				
1	64	**0.292**	0.547	0.502
2	64	**0.269**	0.333	0.307
3	85	**0.260**	0.235	0.158
**KIND OF STATEMENT**				
Specific	114	**0.222**	0.279	0.095
Generalizable	99	**0.315**	0.466	0.355

Performers' levels of agreement with each other are in bold.

A first observation is that by conventional interpretation the kappas for the players' ratings indicate only moderate to fair agreement (Landis and Koch, 1977) or fair to poor agreement (Fleiss, 1981). How different are these levels of agreement from what would occur by chance? The kappa statistic is intended to account for chance agreement, but given that our usage of kappa is unusual we simulated what chance agreement would look like for this many raters, statements, and categorizations of statements by creating two data sets of 217 simulated ratings by three raters. These data sets included randomly generated integers between 1 and 3 with equal likelihood of occurring, to simulate our three levels of rating. We then repeated all the kappa comparisons summarized in Table 3 on these data sets. All kappas using this procedure ranged between -0.101 and +0.110, with the majority close to 0. All but two of the kappa values in Table 3 are greater than 0.110; clearly the agreement among our participants was greater than chance.

Second, consistent with the endorsement findings (Figure 3), we see little evidence that the players have privileged agreement relative to the outside listener: in general kappas were lower for sax-pianist agreement than for sax-listener or pianist-listener agreement. Statements made by the sax player generated the highest agreement (whether endorsement or dissent) across the board, while agreement between the sax player and the outside listener about the listener's statements was notably low. The sax player and pianist agreed most about statements about Performance 1, as did both players with the outside listener. The generalizable statements elicited higher agreement by all three pairings than the specific statements. But all in all the level of agreement was not particularly high.

Because a neutral rating by one party could potentially be interpreted as (lukewarm) agreement with the other party's endorsement or dissent, we also calculated Cohen's kappa under this more generous assumption. Table 4 shows kappas calculated using an alternative set of three rating categories: endorsement (either both parties rated a statement 4 or 5 on the 5-point scale or one endorsed and the other was neutral), both neutral, and dissent (either both parties rated a statement 1 or 2 on the 5-point scale or one dissented and the other was neutral).

**Table 4:** **Inter-rater agreement (Cohen's kappa) calculated assuming that neutral ratings by one party constitute agreement with the other's endorsement or dissent (Categories with too few statements for kappa to be calculated are not reported)**.

	**Number of statements**	**Sax-pianist agreement**	**Sax-listener agreement**	**Pianist-listener agreement**
Overall	217	**0.501**	0.463	0.414
**STATEMENT MADE BY**				
Saxophonist	62	**0.543**	0.640	0.447
Pianist	90	**0.373**	0.446	0.304
Outside listener	50	**0.662**	0.074	0.199
**PERFORMANCE THAT WAS RATED**				
1	64	**0.412**	0.595	0.554
2	64	**0.606**	0.510	0.438
3	85	**0.480**	0.267	0.264
**KIND OF STATEMENT**				
Specific	114	**0.500**	0.331	0.165
Generalizable	99	**0.471**	0.556	0.496

Performers' levels of agreement with each other are in bold.

Under this assumption, the kappa levels are notably higher, by traditional interpretation moving more into the “moderate” territory, with a few that Landis and Koch (1977) would call “substantial” (sax-pianist agreement about performance 2 and about statements by the outside listener, sax-listener agreement on statements by the sax player). In this way of looking at the ratings, there is now more evidence for privileged agreement among the players relative to the outside listener, in that there are higher kappas for the players with each other than with the outside listener overall, for statements made by the outside listener, for statements about Performances 2 and 3, and for specific (but not generalizable) statements. Nonetheless, the fact that the sax player and pianist agreed most about the statements made by the outside listener and less about the statements that they themselves had made, and least about statements made by the pianist, is not consistent with a simple version of players' privileged understanding.

Otherwise, this more generous view of inter-rater agreement is consistent with the Table 3 view in several ways. Statements made by the sax player still elicited particularly high agreement between sax-listener and pianist-expert. The agreement between the sax player and the outside listener about the listener's statements was again notably low. But in this view, the players agreed with each other most about Performance 2 relative to the others, and there is less of a difference in how they rated specific and generalizable statements (although they still agreed more with the expert about generalizable than specific statements).

**Figure 4:**
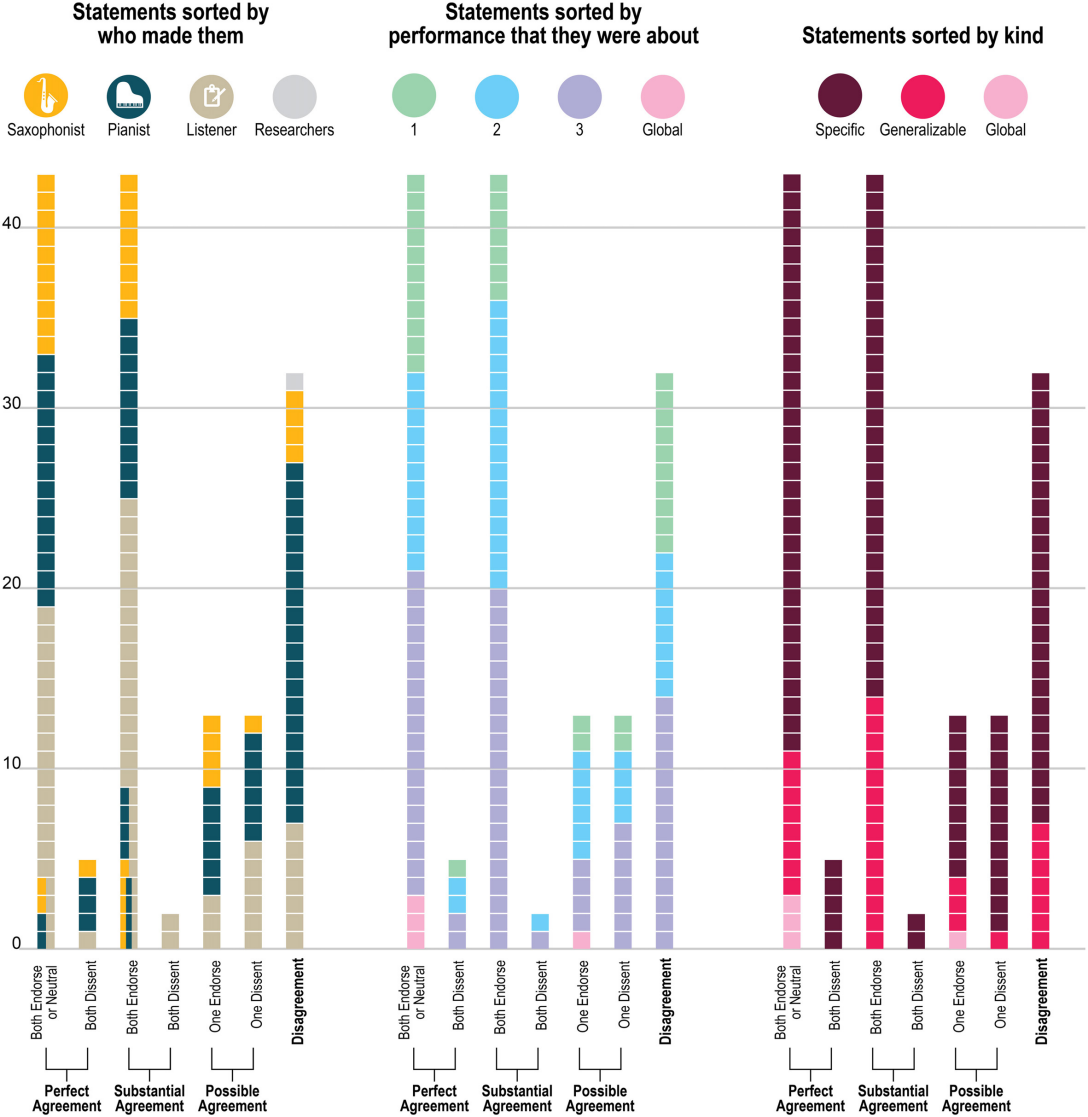
**Distribution of players' agreement for the 151 unique statements**. “Perfect agreement” means that both players gave exactly the same rating (from 1 to 5 on the 5-point scale); “substantial agreement” means that both players' ratings differed by only 1 point and they were either both endorsements or both dissents; “possible agreement” means that one of the players' ratings was neutral and the other's was not, which could either be seen as agreement or disagreement; and “disagreement” means that ratings differed by 3 or 4 rating points.

Figure 4 presents a closer look at the distribution of the statements about which the players agreed—both endorsing or both dissenting—and disagreed. (The figure includes ratings of all 151 statements about the original recordings to which each statement referred, but leaves out the ratings for the “generalizable” statements applied to the other two recordings; ratings for the generalizable statements are presented in **Figure 6**). As Figure 4 demonstrates, the disagreements (as well as the agreements) came from statements by all three sources (saxophonist, pianist, outside listener); from statements about each of the three performances as well as from the 4 global statements (e.g., “My partner listens to a lot of the stuff that I listen to”) and from statements that were specific to only one performance and generalizable across performances. The fact that the agreements and disagreements do not all come from the same source or the same performance suggests that the level of agreement observed here applies generally across this pair's entire performance experience, as opposed to only a particular moment, and that no party's characterizations were uniformly endorsable.

Figure 5 presents a yet more fine-grained view of the 151 statements included in each level of agreement, and Figure 6 presents a similar view of ratings of the 33 generalizable statements across the 3 performances. As one can see, statements with the most disagreement included not only judgments of quality of one party's performance (“The pianist's opening was excellent”; “The pianist's chord at about 1:23 didn't work”) or the ensemble (“This was not the best performance”), but also assessments of the nature of the collaboration (“During these two choruses starting at about 1:22 the sax hears and uses the pianist's substitutions”) and—surprisingly—even basic musicological facts about what happened (“At about 2:21, the sax started a phrase on B flat”).

**Figure 5A:**
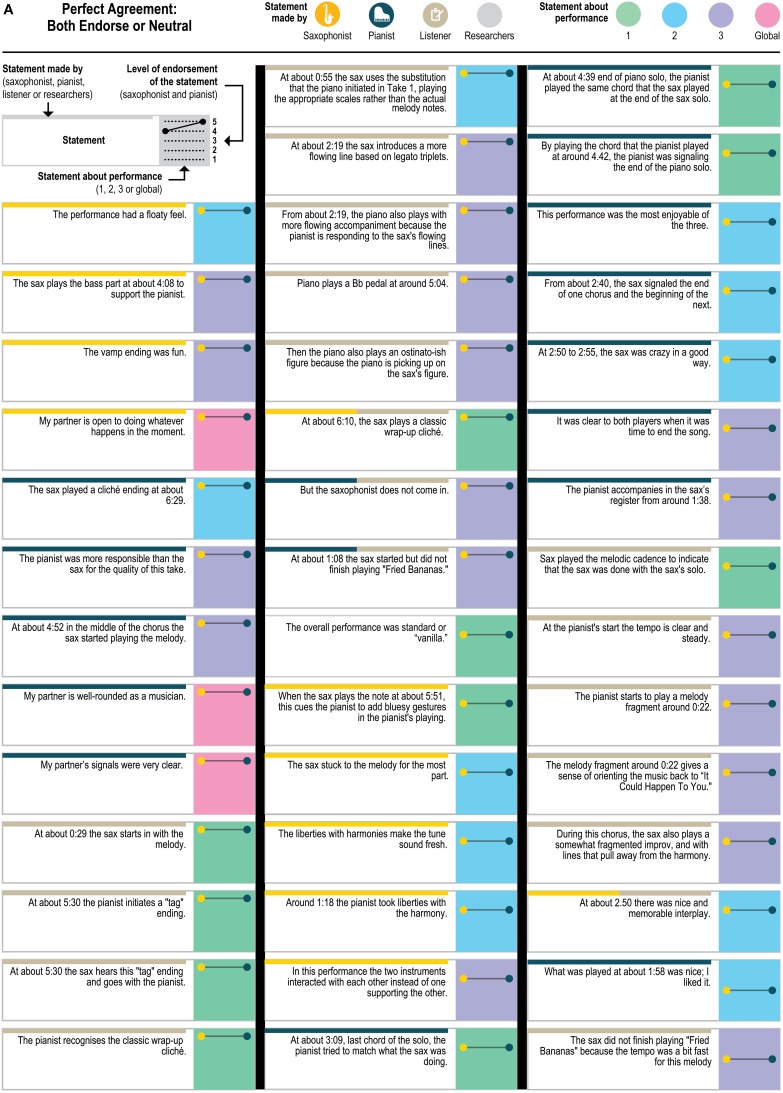
**Statements with perfect agreement where both players endorsed the statement or were neutral**.

**Figure 5B:**
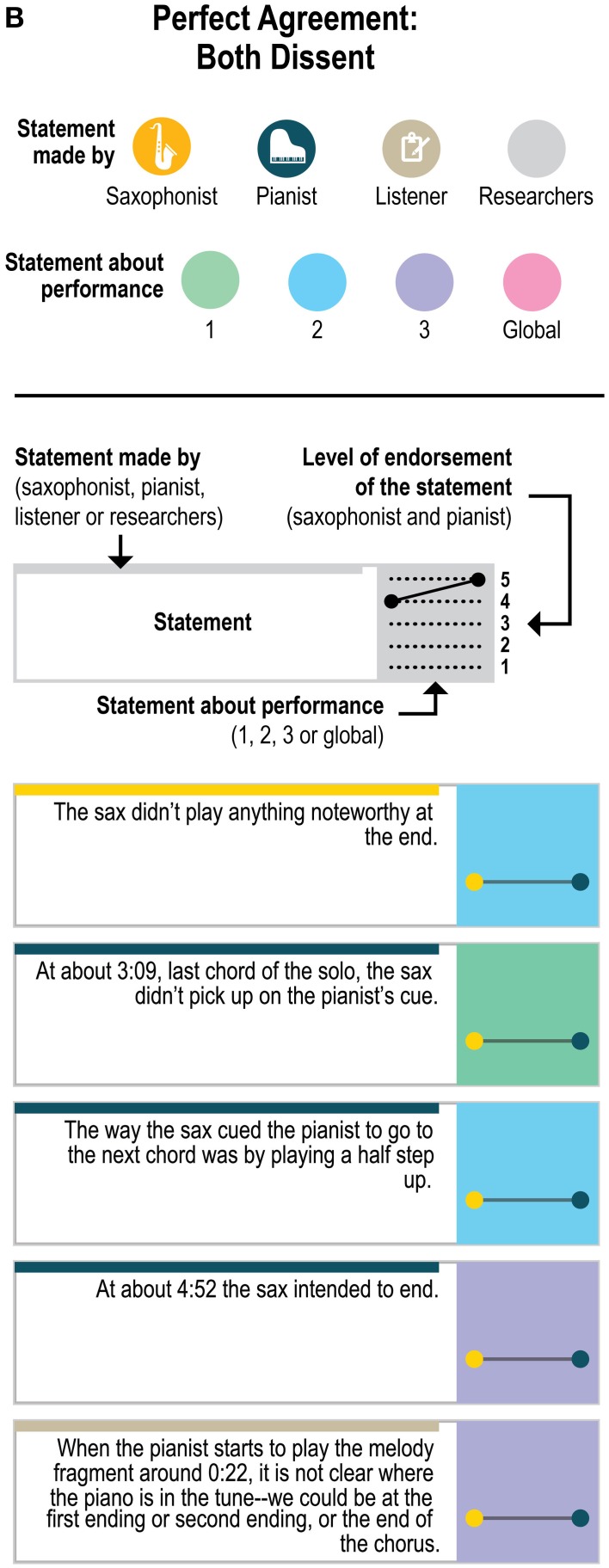
**Statements with perfect agreement where both players dissented**.

**Figure 5C:**
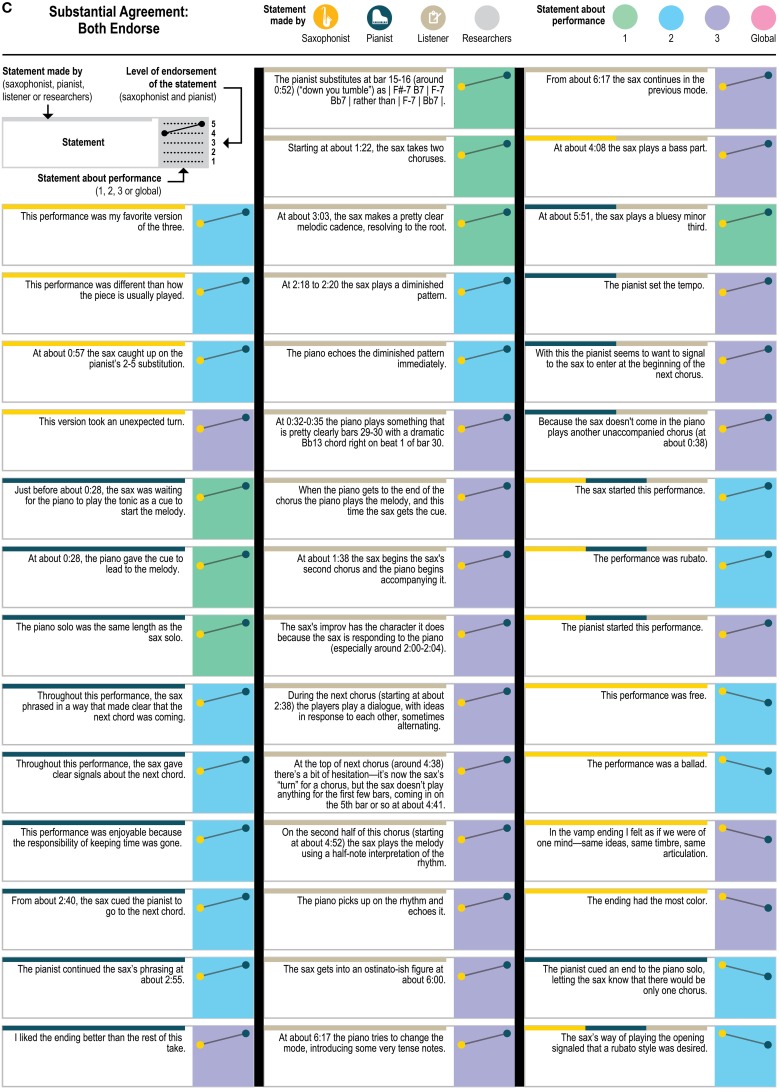
**Statements with substantial agreement (ratings different by only 1 point) that both players endorsed**.

**Figure 5D:**
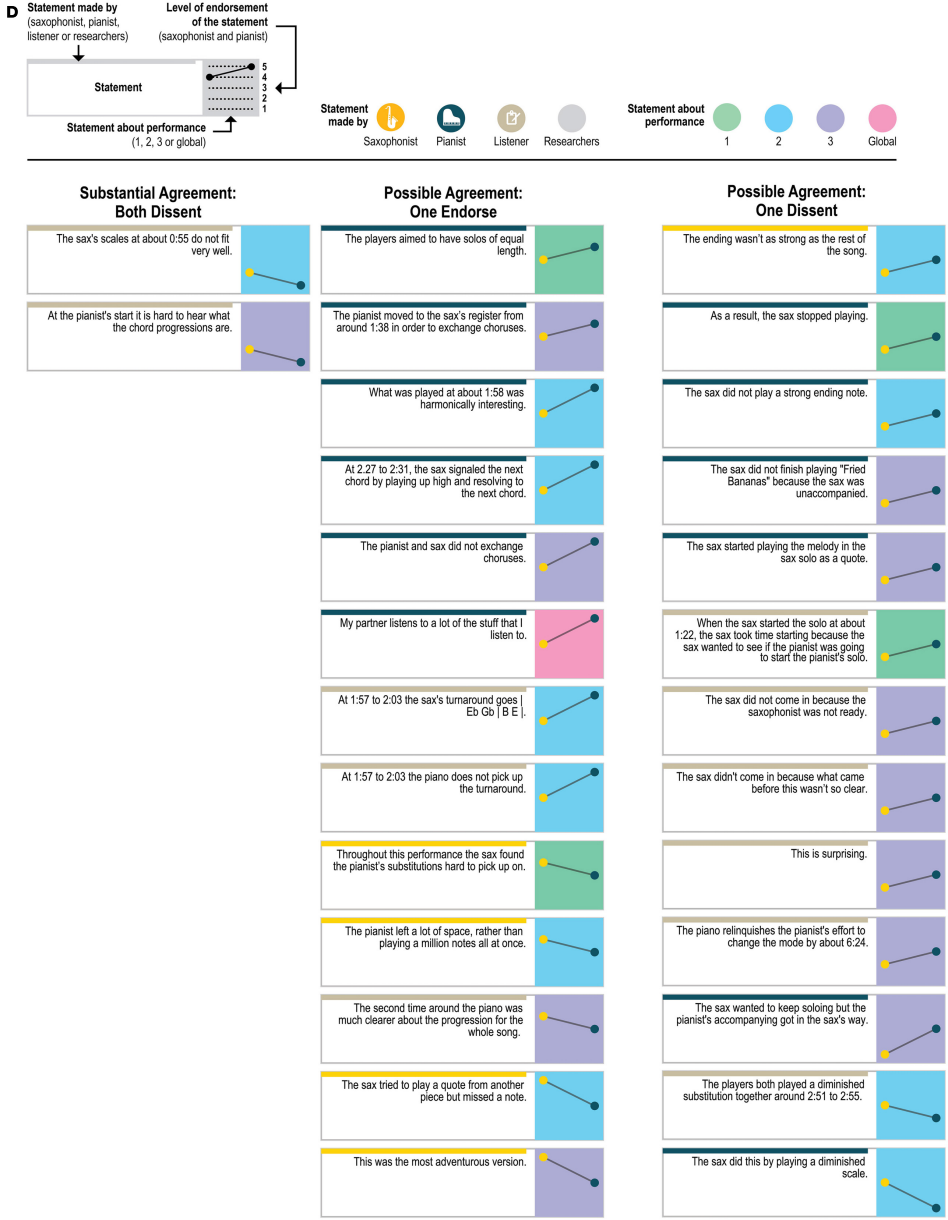
**Statements with substantial agreement (ratings different by only 1 point) where both players dissented, and statements with possible agreement (one player's rating was neutral and the other's was not)**.

**Figure 5E:**
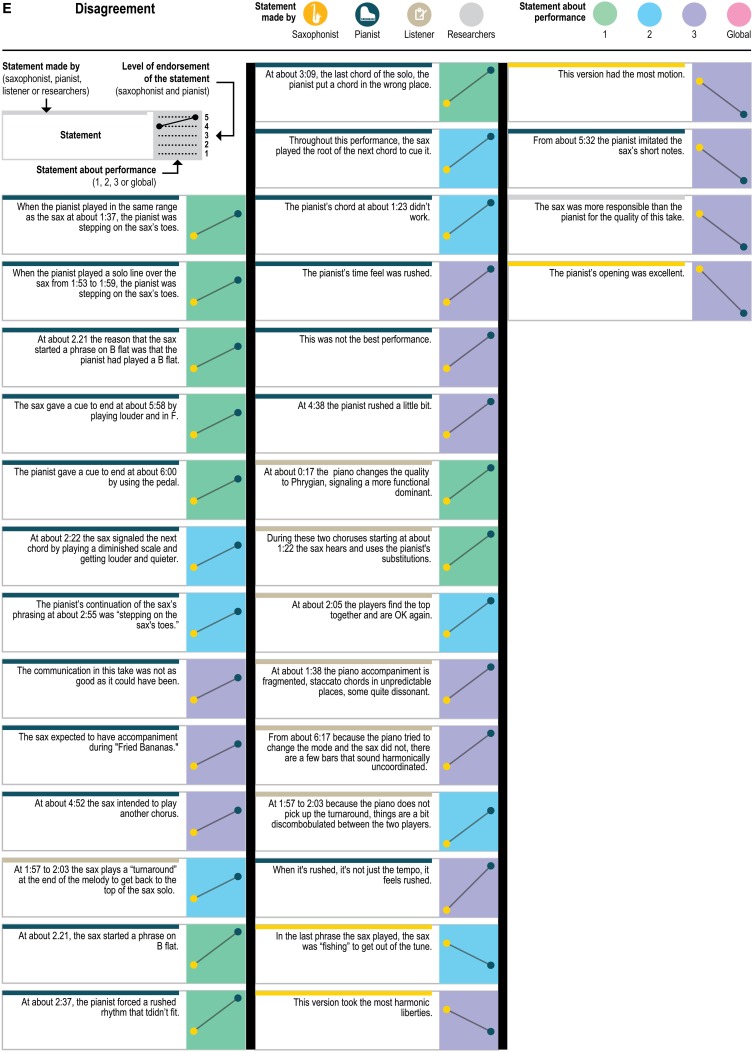
**Statements with disagreement (ratings differed by 3 or 4 rating points)**.

**Figure 6:**
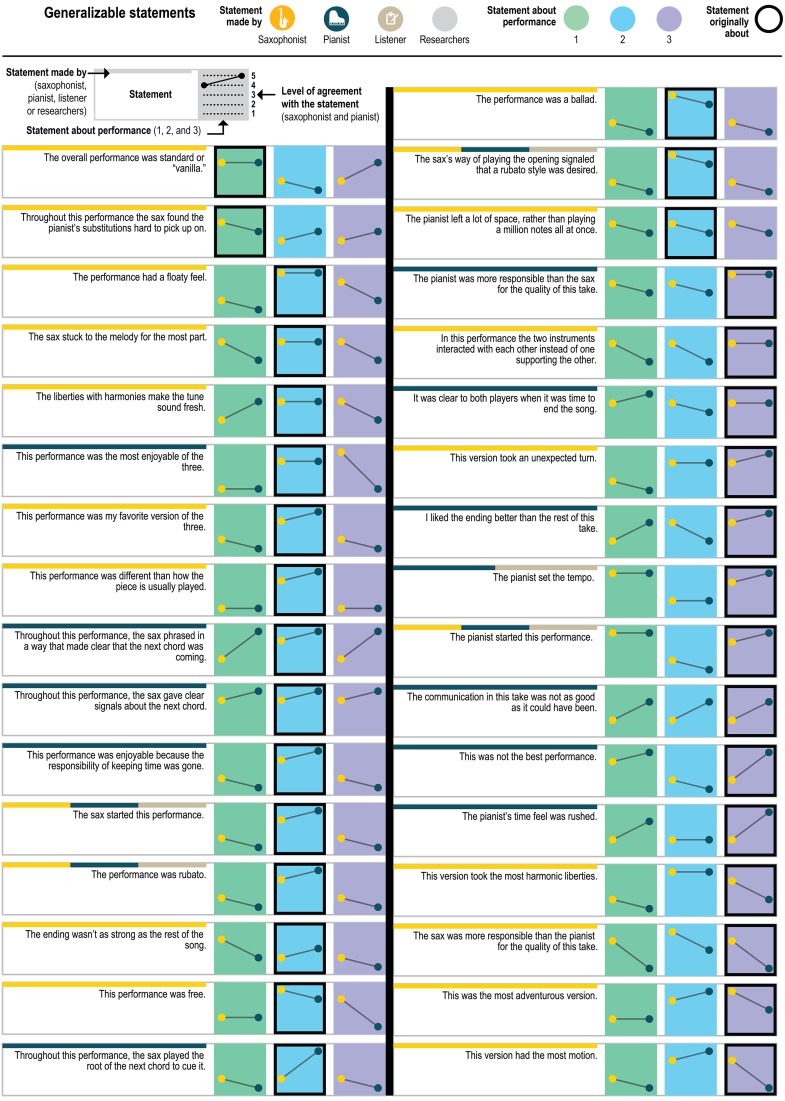
**Players' ratings of the generalizable statements for all three recordings**.

#### Elaborations about disagreements

Given the generally high level of musicianship in the pair (supported by the expert listener's assessment), we were surprised at some of the statements on which they disagreed, particularly those about musicological facts. In the individual follow-up with each player, we asked them to give more detail about their dissents for the 35 statements on which they clearly disagreed with the other (by 3 or 4 rating points)—28 for the saxophonist and 7 for the pianist. For example, was the sax player's dissent with “At about 2:21, the sax started a phrase on B flat” because he disagreed that this happened at all, that there was a new phrase, that the phrase started on B flat, or that it happened at 2:21? Depending on where the dissents originate, we would come to quite different conclusions about this pair's shared understanding.

Table 5 presents the full text of the elaborations. Our interpretation is that 7 of the apparent disagreements in rating may not reflect true disagreement; they may be explainable as actually being about differing interpretation of the words in the statements, or to suggest more agreement than the rating implied. A larger number of disagreements (17) strike us as resulting from differing ideological stances about the nature of intention and causality in jazz improvisation; these disagreements may or may not reflect substantive differences in shared understanding during the course of playing. But a substantial remaining number (11) seem to reflect true differences in judgment about the quality of the performances and about who was responsible.

**Table 5:** **Elaborations by players on why they had dissented**.

**Statement**	**About performance**	**Dissenter**	**Dissenter's elaboration**
**POSSIBLY NOT DISAGREEMENT**			
Throughout this performance, the sax phrased in a way that made clear that the next chord was coming.	1	Saxophonist	As we were playing each example I let the piano establish the harmonic progression and chose to follow rather than lead.
Throughout this performance, the sax phrased in a way that made clear that the next chord was coming.	3	Saxophonist	As we were playing each example I let the piano establish the harmonic progression and chose to follow rather than lead.
During these two choruses starting at about 1:22 the sax hears and uses the pianist's substitutions.	1	Saxophonist	I only disagree here because it took me a little longer than I would have liked to figure out those substitutions. I don't remember “playing” them, but rather fumbling over them at first. I knew something interesting harmonically was happening, I just couldn't figure it out fast enough. Although it is a very simple and very well-used technique.:)
The pianist gave a cue to end at about 6:00 by using the pedal.	1	Saxophonist	… it's hard to make any definitive claims as to what was being done in an improvisation, but perhaps the use of a pedal was a clue to me to wrap it up.
At about 1:38 the piano accompaniment is fragmented, staccato chords in unpredictable places, some quite dissonant.	3	Saxophonist	What seems dissonant to one person could very well sound beautiful and melodic to another. Taste is subjective.
This version had the most motion.	3	Pianist	The word motion is kind of abstract… I was thinking more about the overall arc of the tune? That means we start in one place and we go through certain things and then we end in another place, and I consider that is like a good motion for a whole song. And I think that Take 3 didn't have that as strongly as the other ones… That's just a matter of what we call motion in a tune.
From about 5:32 the pianist imitated the sax's short notes.	3	Pianist	I didn't catch that from listening to it right now, I don't know. It's kind of an open part and he plays something but it almost sounds like I was looking in the wrong spot… he's not really playing the short notes, I think from what I just heard.
**IDEOLOGICAL DIFFERENCE**			
Throughout this performance, the sax played the root of the next chord to cue it.	2	Saxophonist	Playing the root throughout would not be something I could see myself doing. Maybe a for a few measures if there was uncertainty of what we were actually playing, but since that was not the case here I don't think I would have to do that very much.
At about 0:17 the piano changes the quality to Phrygian, signaling a more functional dominant.	1	Saxophonist	When I'm in the moment, I don't have the luxury nor the interest to think to myself “Oh, he went Phrygian there… so I'll play THIS scale.” It doesn't work like that for me. If I was able to hear that texture and react to it in the moment, great but if not great. 0:17 is already in the past and we're on to other music.
At about 2.21, the sax started a phrase on B flat.	1	Saxophonist	This seems like a very subjective observation. I could have used a Bb in my line but who knows if I conscientiously made a move to “Bb.”
At about 2.21 the reason that the sax started a phrase on B flat was that the pianist had played a B flat.	1	Saxophonist	Again, it's all subjective. Maybe I did play a Bb off of the piano's Bb, but if we were to do this tomorrow I might play another note altogether.
At about 2:37, the pianist forced a rushed rhythm that didn't fit.	1	Saxophonist	It's hard to imagine “rushing a rhythm in to fit.” There was elasticity in the takes, some more than others but when playing duo like this and not married to a bass player and/or drummer “time” is all relative.
At about 3:09, the last chord of the solo, the pianist put a chord in the wrong place.	1	Saxophonist	Who is to say what is “wrong” or what is “right”? There is no wrong or right, there just is. That is JAZZ. Just because it might have been unexpected does not make it “wrong.”
The sax gave a cue to end at about 5:58 by playing louder and in F.	1	Saxophonist	Again, it's hard to make any definitive claims as to what was being done in an improvisation.
The pianist's chord at about 1:23 didn't work.	2	Saxophonist	Who's to say what works and what doesn't work?
At 1:57 to 2:03 the sax plays a “turnaround” at the end of the melody to get back to the top of the sax solo.	2	Saxophonist	Labeling and analyzing of playing is all and well, but it's not how I think when playing. It's a sound that I will use at the end of a chorus to get to the top. It may or may not be a “turnaround” but again, who is making these calls?
At 1:57 to 2:03 because the piano does not pick up the turnaround, things are a bit discombobulated between the two players.	2	Saxophonist	If anything, when things seem “wrong” or “discombobulated” it often is a time for the players to really explore the limits of improvisation where they are forced to abandon their clichés and licks and find their way back to calmer waters but it is so hard to say “wrong.”
At about 2:05 the players find the top together and are OK again.	2	Saxophonist	Tied into [previous elaboration], when improvising in a duo there is freedom harmonically, melodically, and rhythmically. There are no “rules” and the players determine when and where to “get off” without any predetermined thoughts or plan.
At about 2:22 the sax signaled the next chord by playing a diminished scale and getting louder and quieter.	2	Saxophonist	Again it's too hard to say for sure that just because a certain scalar passage was played that it determined or signaled a next chord. Even when playing in “free” time, if you are playing a “tune” then there is a certain harmonic blueprint that no matter how you manipulate or extrapolate it, it is always going on subconsciously in your brain and regardless of what liberties are taken in the moment, players will organically gravitate toward moments that they can “ground” themselves back in.
The sax expected to have accompaniment during “Fried Bananas.”	3	Saxophonist	How can anybody say for sure what another person was expecting or not? It doesn't make sense. Since this is all happening on the fly, the players need to be ready for anything.
At 4:38 the pianist rushed a little bit.	3	Saxophonist	I don't believe that one can “rush” when there is no absolute “time.” Flexibility in time is what gives improvisation in this context its sense of freedom.
When it's rushed, it's not just the tempo, it feels rushed.	3	Saxophonist	What “feels” rushed to a listening or someone analyzing tapes most likely doesn't “feel” the same to the players. In the context of a duo performance, any player has the authority to change tempos when and if they feel like it. But since there are no other “time keepers” (bass or drums) to lock the performers into “tempo,” the time can rush or slow down at their liking.
At about 4:52 the sax intended to play another chorus.	3	Saxophonist	How can one be so sure that the sax player “intended” to play another chorus? There have been plenty of times in my performance career when improvising you decide at the last minute—“Ok, that was enough. Do I really NEED to play ANOTHER chorus?” Most often, I decide that I do not need to play another chorus and just get out of the tune.
From about 6:17 because the piano tried to change the mode and the sax did not, there are a few bars that sound harmonically uncoordinated.	3	Saxophonist	More issues with what sounds “right” or “wrong.” When improvising it may take some time for the players to get on the “same page” but it is the search and the journey to find where they are going that is the great joy of improvisation.
**DISAGREEMENT IN JUDGMENT AND EXPERIENCE**			
The pianist's time feel was rushed.	3	Saxophonist	The pianist had a really great time feel throughout each of the three takes. At no time did I feel he was rushing.
This was not the best performance.	3	Saxophonist	I think that I felt that this take was “better” than other takes we did.
The communication in this take was not as good as it could have been.	3	Saxophonist	I felt like communication was great throughout each of the three takes. That's the beauty of using your ears and playing through improvisation.
When the pianist played in the same range as the sax at about 1:37, the pianist was stepping on the sax's toes.	1	Saxophonist	I felt that the pianist was very tasteful throughout and at no time felt like my toes were being stepped on. Even in the same range. If anything, it created a unique texture that is often overlooked because of the range of the piano.
When the pianist played a solo line over the sax from 1:53 to 1:59, the pianist was stepping on the sax's toes.	1	Saxophonist	Again, to me I did not feel like my toes were being stepped on throughout the recording.
The pianist's continuation of the sax's phrasing at about 2:55 was “stepping on the sax's toes.”	2	Saxophonist	There was no stepping on the sax player's toes.:)
This performance was the most enjoyable of the three.	3	Pianist	Overall communication there were the most things happening that were not together… Everybody was expecting the other guy to do something else the most times out of all the three takes… the first two takes were much better in that sense.
This version took the most harmonic liberties.	3	Pianist	Take 2 was the one with the most harmonic liberties, because it was free of form, it was out of time… more than the first and third take as far as the harmony involved.
The sax was more responsible than the pianist for the quality of this take.	3	Pianist	About the saxophone… I'm saying he wasn't the one leading… I started playing by myself, I was expecting him to go certain places and… he didn't go to some of them but I think… I couldn't expect him to follow me in that sense. But the idea of me leading the take is more true than my following him.
In the last phrase the sax played, the sax was “fishing” to get out of the tune.	2	Pianist	When you say somebody's fishing for the ending that means… that it's not together and they are not sure where they are or what's supposed to happen but from what I heard that sound is really clear. But on the other hand I think I was kind of leading/forcing… that sort of ending and he was kind of following what I was doing… that particular phrase at least was more him following my harmony.
The pianist's opening was excellent.	3	Pianist	In general I tend to judge and criticize myself a lot so… I don't like to hear “excellent” along with my intro.… I had this idea in mind to play one chorus by myself, and then the other guy is going to play a chorus, and we're kind of going to trade choruses. But he didn't come in on the second chorus and it's kind of obvious because there weren't a lot of chances of his predicting exactly that. That's a pretty far out thing to predict when you're not talking or seeing. I think that's why I cannot say it was excellent because I could either have made it more obvious for it to work or think of a different idea. It's not about what I'm actually playing for the first chorus when I'm playing by myself. I jumped right into the song, it's not a real intro actually.

For the elaborations we classified as reflecting possible non-disagreement, in some cases they seemed to reflect alternate interpretations of the wording of the statements. For example, the saxophonist's disagreement with “At about 1:38 the piano accompaniment is fragmented, staccato chords in unpredictable places, some quite dissonant” seems to stem from an interpretation of the word “dissonant” as meaning “not beautiful,” as opposed to simply a neutral musicological meaning with which he may well have agreed. Similarly, the pianist's disagreement with “This version had the most motion” stems from his sense of the word “motion” as referring to the overall arc of an improvisation, as opposed to a description of tempo or propulsion. The saxophonist's disagreement with “During these two choruses starting at about 1:22 the sax hears and uses the pianist's substitutions” does not reflect a belief that he never used the pianist's substitutions at all during these two choruses, but a regret that he didn't do it sooner. Another disagreement (“From about 5:32 the pianist imitated the sax's short notes”) may well have resulted from the pianist's not agreeing that the short notes happened at 5:32, as opposed to their not happening at all or his not having imitated them.

Another set of elaborations, all by the saxophonist, seem to reflect a stance that rejects straightforward claims about causality and the definitiveness of intentions in jazz improvisation—or at least an ideology about what kinds of claims are appropriate to make in an interview about improvisation. It is unclear whether these disagreements reflect a real difference in the mental life of the players during the performances. The saxophonist rejects on ideological grounds the idea of “rules” or “right and wrong” or “what works and doesn't” in jazz, as well as any sense of inevitability about what happens in an improvisation, while the pianist and expert use terms that suggest that certain moments worked and others didn't. He also clearly rejects the idea that he may have been carrying out musicological analyses while playing, as this was not part of his conscious experience along the way.

There are hints in the saxophonist's elaborations of a more complicated stance, in that he does assume that moments can feel “wrong” or “discombobulated”; that there was something to figure out when he “couldn't figure it out fast enough”; and he refers to a “harmonic blueprint” that constrains what players do in improvisation, despite his resisting the idea of “predetermined thoughts or plans.” Although his elaborations reflect the avoiding-blame “yes-and” approach that is essential to improvisation (e.g., Berliner, 1994; Crossan, 1998; Weick, 1998), he also is self-critical in not having picked up on substitutions quickly enough. And although he resists endorsing statements that assign intentions or conscious musicological analysis to any player (including himself), he also describes intentional thoughts in the moment of playing, even if those intentions are fleeting and inconsistent and subject to revision. But again, these differences in how the players articulate their relationship with musical intentions may not reflect a true difference in shared understanding during their performance, but rather a different stance on how one talks about jazz improvisation.

Perhaps more telling are what seem to be real disagreements in judgments about the performances and their experience. The players clearly disagree about the quality of Performance 3 on several fronts. The pianist found it to be the least successful performance, while the saxophonist liked this performance best and found it most interesting and liveliest. The saxophonist considered Performance 3 to have taken “harmonic liberties” while the pianist considered it not to have, and the saxophonist considered the players' communication during Performance 3 to have been successful, while the pianist thought that they had not been together and that “everybody was expecting the other guy to do something else.” The pianist considered himself more responsible for what he found didn't work in Performance 3, for example, attributing the fact that the saxophonist did not come in as the pianist had expected at the end of his opening chorus to his not having provided clear enough signals; in contrast, the saxophonist did not rate the pianist as more responsible for the quality of the performance. More generally, the saxophonist outright rejected the pianist's self-criticisms, in particular the pianist's statements that he had been “stepping on the sax's toes” and that he had been rushing, and considered the overlapping-register moves by the pianist to have “created a unique texture.”

Intriguingly, the saxophonist assumes that the statement about the pianist's part feeling rushed must have come from an outsider rather than his partner, reflecting an implicit theory that co-performers will share judgments that outsiders do not. But this statement turns out actually to have been made by the pianist. As we see more generally, the data do not support privileged understanding between the players, relative to this outsider.

## Discussion

### Findings and method

Taken together, the findings suggest less than fully shared understanding between these two performers. Immediately after the performances they did not generate many of the same statements to characterize what had happened in their performances and how they had experienced them—in fact, there were no statements that they both generated that the outside listener didn't also generate. Two months later, their endorsement of the other's (anonymized) statements was notably less than their endorsement of their own or the outside listener's statements. Quantitatively, their agreement with each other's ratings (kappa) was better than chance, but at best moderate and only occasionally substantial if one takes a generous interpretation of “agreement.” And their agreement with each other was not strikingly greater than with the outside listener, suggesting that they did not share privileged understanding relative to this listener.

How important are these differences? In one sense, the extent of agreement that we found could be seen as impressively high relative to the radical difference that could have been observed; the ways that the ratings differ could be seen as providing a profile of each player's relative weighting or valuing of characterizations that they both agree with overall. In another sense, the differences can be seen as more substantial. Focusing on differences in judgment of the performances (as opposed to ideological or stylistic differences in talking about improvisation, or differing interpretations of terms in the statements), the performers differed substantially in their judgment of the quality of one of the performances (Performance 3). They expressed different judgments of responsibility for what had happened and especially who was leading; they had different musicological judgments about the harmonic freedom of that performance, and they expressed differences in relative enjoyment of the performances. In our view, the overall level of agreement and the particular sites of disagreement combine to demonstrate that substantial differences in understanding can occur in improvisation of the quality observed here.

The ideological differences in talking about improvisation were also notable: how intentions were described and assigned, whether one party could be assigned responsibility for what happens jointly, and how much is “predetermined.” It is unclear whether this reflects real differences in mental life or experience while playing, as opposed to varying judgments about what was appropriate to say in this interview setting; in the continuum from “anything goes, no right or wrong” to “not everything goes” discourse, the two players clearly fall in different places. Nonetheless these different orientations suggest different levels of comfort with assigning responsibility and attributing causality in jazz improvisation.

Of course, this case study included only one pair of performers and one outside expert listener, and they played in a particular setting without seeing each other (more like in a recording studio than in many rehearsal and performance venues). How and whether these findings would replicate with other performers in the same genre, performers of different ages or different musical or cultural backgrounds, performers on different instruments, or performers in different genres, and with outside listeners with different expertise, is unknown. And whether the findings would look different if the performers could have seen and talked with each other, and thus relied on visual and conversational cues in both the content of their interviews and in making their later judgments, is also unknown. But the method used here—immediate retrospective recall, subsequent ratings of those judgments (one's own, other players' and listeners'), and further probing about those judgments—could easily be applied to these other situations.

The method combines the advantages of quantified comparison that identifies overall patterns with qualitative focus on the content of performers' experience. We of course do not believe that the statements collected under this method represent a pure or full account of these musicians' mental lives during their performance (as with any retrospective recall; see Ericsson and Simon, 1993). Interviews are complex interactive events (see, e.g., Clark and Schober, 1992; Conrad et al., 2014, among many others), and we do not imagine that the statements analyzed here are necessarily the same as what our performers might have said with other interviewers, or that players' reports even immediately after a performance are direct readouts of their mental life.

In fact, we suspect that further probing of the agreed-upon statements might reveal further disagreements—the kinds of “undetected conceptual misalignments” (Schober, 2005) that are a part of ordinary language use (see also Suessbrick et al., 2000). Because conceptual misalignment is likely to be inherent in a method that focuses on the aspects of performers' mental lives that can be articulated linguistically, we see the further probing after ratings as essential to the method. We should also note that the method requires particularly attentive and articulate performers who are willing to reflect on their musical practice, as well as to engage in a complex set of tasks and materials on multiple occasions. We were fortunate that our performers were so thoughtful, so musically able, and so willing to give our unusual performance situation a try; but we suspect that there is a range of articulateness and willingness across performers, and that this method may be suitable for only a subset of performers. We were also fortunate in our outside listener's thoughtfulness and verbal fluency, but we are aware that listeners are likely to range enormously in their ability to listen or articulate in such an informed and thoughtful way.

Despite all these caveats, the interviews, ratings and subsequent probings from this method do create *an* account of performers' mental life, and we propose that they allow new insights into musicians' shared understanding.

### Further questions about shared understanding

Our approach allows us to begin addressing a larger set of questions about shared understanding and the mental processes involved in coordinating musically. As we see it, there is a range of possibilities for how performers' understandings overlap. At the *extreme individualist* end of the spectrum, players follow their own script (notated or improvised) and simply happen to be playing at the same time as the others. The ensemble works because the players are following a shared (or close enough to the same) rhythmic structure, but there would be little else that they need to share in order to manage to coordinate. At the *extreme collaborative* end of the spectrum, all players are fully aware of—tracking—every gesture by the others, and they initiate each of their own musical gestures as a response to the others' gestures. By definition, then, every sounding of each players' notes is a collaborative act, and intentionally so. The overlap of understanding at all levels—musical, conceptual, intentional—would be complete.

The reality must be somewhere in between, and probably ranges between the extremes at different moments within an ensemble performance. There is also likely variability in which end of the spectrum different musicians are on, and for different pieces and playings and genres. As Keller (2008) outlines, how players allocate cognitive resources and divide attention between their own performance and others' in musical collaboration needs much more elaboration: how they anticipate, monitor, and adapt or adjust to their partners. We propose that attention to performers' conscious mental life—how they understand their own and their partners' intentions and gestures during performance—is an important piece of this story, in the spirit of Schütz's (1951) proposals.

Attending to performers' mental life is likely to raise new and complex questions about the nature of responsibility and causality in interdependent music-making, as co-performing is simultaneously an individual and collective process (and improvisation is more collective than presumed by many, as Cook, 2004, argues). There may be tension between the all-accepting “yes-and” approach needed to allow improvisation and the more judgment-based stance of monitoring oneself, one's partners, and the ensemble for taste, technical abilities and limitations, accuracy and direction; musical gestures in an ensemble may have unintended effects depending on partner uptake, and those effects can be desirable or undesirable from the originator's perspective. And the nature of partner monitoring and the mental processes underlying it are less well understood than they need to be, just as they are less than fully understood in linguistic interaction (e.g., Schober and Brennan, 2003). Players must have some sort of ongoing partner monitor and they must generate some sorts of expectations about what their partner will do, or else they would always assume that the other's bloopers or missteps were fully intended; but how elaborate and full-fledged this monitor is unknown, as is how it waxes or wanes, how it depends on familiarity with the partner and the piece, etc.

Attending to performers' mental life also raises new questions about the nature of shared understanding. One question is about the extent to which their overlapping judgments rise to the level of full mutual knowledge or common ground—that is, judgments that musicians not only both hold but *know* that their partners hold (see Clark, 1996, for extensive discussion of the complexities of mutual knowledge). Our inter-rater agreement provides evidence on overlap of judgments rather than on mutual knowledge about those judgments, which is a different question. Another question is how judgments of the sort we have collected relate to the other kinds of judgments players make about each other and the full construction of social context that improvisation requires (Monson, 1996).

Beyond the obvious questions of whether and how these findings replicate in a larger sample of pairs of jazz improvisers playing different versions of this or another standard, or in larger ensembles, we see a number of further questions and testable hypotheses:

- Does understanding align differently in pairs or ensembles with different degrees of overlap in their cultural and musical background? Would players from different generations or jazz performance communities have less shared understanding?
- How does familiarity or experience with a partner affect shared understanding? Do longstanding pairs or ensembles have greater shared understanding than new groups, from years of communicating with each other in rehearsals and performances? Are ensembles with greater shared understanding more likely to persist as ensembles, along the lines inferable from Murnighan and Conlon's (1991) findings about string quartets that stay together over time?
- Do players share less understanding in improvisations that are seen as less collaboratively successful?
- How stable or variable is accuracy of understanding a musical partner's intentions, and how partner-specific? Are some players better at sharing understanding with more partners? Do more musically advanced players (independent of the success of their collaborations) know more about what the other is thinking, and are they more likely to agree on what happened?
- Does shared understanding differ across musical genres? Is it greater or less in more freely improvised music? With notated music, how do players' expectations about what an ideal partner would do or ought to do (based on the notation) affect their judgments?
- How do players' musical goals—discovery or enjoyment, as opposed to preparing for performance or perfecting—affect shared understanding? During a phase of exploring options, are understandings likely to be more or less shared?
- Is shared understanding greater when performers play together live, and can thus affect and react to each other's gestures, than when they play with a recorded track? Is shared understanding better when players can see each other (see Schober, 2006; Duffy and Healey, 2012)?
- How is players' shared understanding more or less privileged relative to listeners with more and less expertise as performers or as listeners, or with musical experience in genres other than that of the performance?
- Does keeping better track of one's partner's intentions lead to better performance? To what extent does *too much* focus on a partner's intentions distract from high quality music-making?

Variants of all of these questions can also be asked in other domains of joint action, which are likely to have different dynamics depending on the medium of interaction (Clark and Brennan, 1991) and the nature of the joint task at hand—dancing, co-writing, conversing, walking. Perhaps surprisingly, these questions have not been extensively explored even in the most frequently studied domain of joint action, conversation, in which the empirical focus has been more on conversational processes, the mental representations underlying them, and what leads to task success than on the extent to which participants understand the interaction and task outcome (successful or not) in the same way.

Despite the many remaining open questions, we believe this case study allows a few substantive conclusions. The quality of the performances combined with the disparities in agreement suggest that, at least in this pair, fully shared understanding of what happened was not essential for successful improvisation. And the fact that the performers endorsed an expert listener's statements more than (many of) their partner's argues against a simple notion that performers' interpretations are privileged relative to an outsider's.

## Author contributions

Both authors contributed extensively to the work presented in this paper.

### Conflict of interest statement

The authors declare that the research was conducted in the absence of any commercial or financial relationships that could be construed as a potential conflict of interest.
